# Societal Costs and Benefits of Treatment with Trastuzumab in Patients with Early HER2neu-Overexpressing Breast Cancer in Singapore

**DOI:** 10.1186/1471-2407-11-178

**Published:** 2011-05-18

**Authors:** Gilberto de Lima Lopes

**Affiliations:** 1 Department of Medical Oncology, Johns Hopkins Singapore International Medical Centre, Johns Hopkins University School of Medicine, 11 Jalan Tan Tock Seng (Level 1), Singapore, (308433), Republic of Singapore

## Abstract

**Background:**

Trastuzumab has revolutionized the way we treat early Her2Neu-positive breast cancer, as it significantly improves disease-free and overall survival. Little is known about the societal costs and benefits of treatment with trastuzumab in the adjuvant setting in Southeast Asia.

**Methods:**

Societal costs (benefits) were estimated as the sum of direct and indirect costs minus benefits in the base case. Direct costs were derived from 4 treatment centers in Singapore (2 private and 2 public, comprising 60-70% of all patients with cancer seen in the island-nation); indirect costs were assessed as the loss of productivity caused by the disease or treatment. Benefits to society were based on extra years of productivity, as measured by GNI per capita, resulting from the quality adjusted life-years (QALYs) saved with the use of trastuzumab as determined in the models by Kurian, Liberato and Garrison.

**Results:**

Incremental costs in Singapore, in 2005 US dollars, were $26,971.05. Average Cost per QALY was $19,174.59 (Median: $18,993.70). Costs (benefits) to society ranged from a cost of $79.42 to a benefit of $9,263.06, depending on the model used (Average benefit: $4,375.89, Median $3,944.03). Sensitivity analysis ranged from a cost of $10,685.00 to a Benefit of US$17,298.79

**Conclusions:**

Treatment with adjuvant trastuzumab is likely to generate net societal economic benefits in Singapore. Nevertheless, the lower range of possible outcomes does not refute the possibility that treatment may actually generate costs. These costs however clearly fall within the usual range of acceptable cost-effectiveness.

## Background

Breast cancer is a major health care problem in Singapore and worldwide. The most common cancer in women in industrialized nations, it accounted for approximately 209,060 cases and 40,230 deaths in the United States in 2010 and 1.38 million cases and 458,400 deaths across the globe in 2008 [1,2]. In Singapore, annually, 1,100 women develop breast cancer and 270 die from the disease [3].

Major advances have been made in the prevention and treatment of patients with breast cancer in the last 2 decades. Currently more than 90% of patients survive for 5 years or greater after their diagnosis, compared to 75% or fewer 30 years ago [1].

Newer anti-cancer drugs have been rationally designed to target a subset of patients with breast cancer: women whose tumours over-express a protein named human epidermal growth factor receptor-2 (HER-2Neu). One of these medications, trastuzumab, a monoclonal antibody, has recently been shown to decrease recurrences and mortality by 50% in patients with early breast cancer treated with surgery followed by chemotherapy, radiotherapy and hormonal therapy when appropriate [4,5]. It is therefore the first targeted agent that may cure patients with breast cancer. Adverse events are usually mild but this new medication comes with a high price tag.

In order to assess if a health care payer (government system, insurance company or self-paying patient) should approve the use of the drug, this study aimed to evaluate the societal costs and benefits of treatment with Trastuzumab in patients with early breast cancer in Singapore and to discuss policy implications.

While there are no clinical studies performed in the Singapore setting to confirm the survival and recurrence prevention benefits seen with trastuzumab in the United States and Europe, available evidence suggests that those results can be extrapolated to the island nation. This is the first study to assess the cost-benefit of trastuzumab in early breast cancer in Singapore [6].

## Methods

### Literature Search

A systematic search of the medical and health economics literature using the MEDLINE/PUBMED and EMBASE databases and the Johns Hopkins University Library system (supplemented by manual reading of review articles in search for further references) revealed three studies which evaluated the cost utility of trastuzumab given concurrently with paclitaxel chemotherapy following 4 cycles of an anthracycline and cyclophosphamide (The most common current standard of care worldwide and in Singapore). Details of the search(es) were as follows, (a). Terms used: trastuzumab, cost-effectiveness, cost-utility, cost-benefit, adjuvant, early breast cancer in MESH terms and with several permutations; (b). Results: trastuzumab AND cost-effectiveness (or cost-benefit) retrieved 50 citations. Review of these citations revealed the three studies included in this paper as mentioned above and also: 11 studies that assessed a strategy of trastuzumab following chemotherapy (and not concurrent and then following as is the current standard in Singapore), several other studies, including budget impact papers, studies of other drugs and several review articles.

### Health Benefits

The three detailed cost-utility analyses published by Liberato et al [7], Kurian et al [8] and Garrison et al [9] form the basis of the health benefits data used in this report as the quality adjusted life year (QALY) benefits for trastuzumab are derived from their models. Other clinical benefits are derived from the 3 clinical trials that set trastuzumab as the new standard of treatment for patients with HER2Neu-expressing early breast cancer: HERA, NSABP B31 and NCCT 9831 [4,5].

Assumptions from each of the datasets: Kurian et al, Liberato et al and Garrison et al can be seen in their original papers and in additional file 1.

In summary they used Markov models to estimate the long term health outcomes, quality of life and costs from treatment with trastuzumab with results presented as incremental cost-effectiveness ratios (ICER), in cost per QALY saved for the following populations receiving adjuvant therapy with or without trastuzumab. In addition, Kurian et al assessed a non-anthracycline-containing trastuzumab (NAT) regimen.

Kurian et al showed that in their base case analysis, treatment without trastuzumab (NT) yielded 9.35 QALYs at a cost of US$133,429, the anthracycline-containing trastuzumab (AAT) regimen yielded 10.77 QALYs at a cost of US$190,092, and the non-anthracycline-containing regimen (NAT) yielded 10.61 QALYs at a cost of US$206,561. Compared with the NT regimen, the AAT regimen yielded an ICER of US$39,892/QALY. The AAT regimen dominated the NAT regimen (meaning that it cost less and was more effective). Liberato et al calculated that adjuvant trastuzumab resulted in a benefit of 1.34 life-years and 1.54 QALYs with an incremental cost of €15,476 (US$20,211) for adjuvant trastuzumab and an incremental discounted cost effectiveness of €14,861/QALY (US$18,970/QALY). Garrison et al determined that treatment with trastuzumab cost an additional US$44,923 and had an expected gain of 1.70 QALYs.

### Costs

All costs and benefits are incremental, i.e., they only include the added expenses or gains associated with the use of trastuzumab beyond those related to other treatments. The author used Microsoft Office Excel 2007 (Microsoft Corporation, Redmond, WA, United States of America) to determine net present values (NPV) with discounting as described under general assumptions below.

Direct costs were obtained with a private patients' perspective through a survey of treatment charges for the hypothetical base case at 4 cancer centers in Singapore: two government hospitals and 2 private institutions. Together, these institutions provide treatment to 60-70% of patients with cancer treated in Singapore (Personal communication).

These costs are expressed as a weighted average per number of new patients seen yearly. The weights were: 4,200 for restructured hospital number 1; 900 for restructured hospital number 2; 600 for private medical centre number 1; and 200 for private medical centre number 2. These are the estimated numbers of new patients with cancer seen yearly at each institution.

Incremental direct costs comprised all medication and infusion expenses related to treatment with chemotherapy and trastuzumab in the adjuvant setting. Costs associated with primary local, regional (radiation and surgery) and systemic (chemotherapy) treatment were not included. To determine the base case dose of trastuzumab, the author calculated the median and mean weight of a sample of 40 consecutive women receiving chemotherapy at one of the hospitals to be 48 kg (Data not shown).

The following expenses, based on medical oncologist opinions and discussions with the business offices of each medical centre, were added to the calculation of incremental direct costs associated with trastuzumab per patient: Her2Neu testing (assuming all patients were tested for immuno-histochemistry and 30% for fluorescence in situ hybridization), trastuzumab for 1 year (drug and infusion costs), 9 extra consultations with medical oncologist, 4 echocardiograms to monitor heart function, and the cost of treatment of cardiac toxicity (incidence estimated at 4%, cost included 3-monthly cardiology visits and medications for 1 year). The costs of excess cancer recurrence (local and distant) for patients who did not receive trastuzumab (i.e., the direct benefits) were subtracted from the costs of trastuzumab as they represent a cost saving in favour of the treatment.

The cost of local recurrence included fees for surgery and hospital stay as well as treatment with trastuzumab for 1 year multiplied by its predicted frequency. Standard of care for patients who progress systemically on trastuzumab included treatment with (a) capecitabine and lapatinib, or (b) capecitabine and trastuzumab. Once patients progressed they received trastuzumab and lapatinib in combination (c). The estimates assumed that patients received 9 months of treatment with (a) or (b) followed by 3 months of treatment with (c)

Indirect costs were assessed as the loss of productivity derived from absence from work for treatment (Derived from Gross National Income per capita data). In addition, the cost of transportation for each treatment was estimated based on median mass transit fare in Singapore.

### Benefits

Gross national income per capita (GNI/capita) was the measure of individual productivity used to calculate the benefits of treatment. Benefits to society were based on extra years of productivity, resulting from the quality adjusted life-years (QALYs) saved with the use of Trastuzumab. As these were determined independently by Liberato et al [7], Kurian et al [8] and Garrison et al [9], the results section presents 3 sets of benefits, one from each dataset.

### Calculation of Societal Costs and Benefits

Societal costs (benefits) were estimated as the sum of direct and indirect costs minus benefits. To account for variability, 95% Confidence Intervals were calculated based on the 95% confidence intervals for QALYs gained from the datasets by Kurian and Liberato and the range of possible QALYs are depicted for the results derived from Garrison's [7-9].

### Other Assumptions

#### General Assumptions

This report uses GNI per capita estimates from the International Monetary Fund for 2005 [10] values at purchasing power parity. Euros and Singapore dollars were converted to United States Dollars at the median exchange rate in 2005 [11]. All future benefits and costs were discounted at a 3% rate and represented as 2005 United States Dollars complying with the guidelines produced by the US Public Health Service Panel on Cost-Effectiveness in Health and Medicine [12].

Gross National Income per capita was derived from the International Monetary Fund and World Bank data for 2005 at purchasing power parity [13,14].

United States Dollar to Euro median exchange rates were derived from the European Central bank data [11]. Singapore Dollar to United States Dollar median exchange rates were obtained from the Monetary Authority of Singapore [15].

## Results

Direct Costs and Benefits of Trastuzumab in the Treatment of Early Breast Cancer in Singapore

Table 1 shows the costs associated with the use of trastuzumab in the treatment of early breast cancer in Singapore for the base case. Total incremental patient costs without subsidies at the 2 government restructured hospitals were S$ 51,040.63 and S$ 35,462.94 (government hospitals have different purchasing contracts for many cancer drugs, likely explaining the difference between them). Not surprisingly costs were higher at the 2 private medical centres, at S$ 62,769.82 and S$ 70,376.73. (All in 2009 Singapore dollars)

**Table 1:** Incremental Direct Costs and Benefits Associated with Trastuzumab in the Treatment of Early HER2neu-Positive Breast Cancer in Singapore

Cost Element				Hospital		
			**PMC 1**	**PMC 2**	**GRH 1**	**GRH2**

HER2neu testing			615.10	410.50	133.00	322.14

Trastuzumab						

	Direct Drug Costs		82,798.74	74,250.00	48,656.88	64,152.00

	Administration Costs		4,500.00	3,870.00	1,289.70	1,908.00

Extra Visits with Oncologist			1,170.00	1,498.00	350.00	749.00

Cardiac Monitoring			1,300.00	1,572.00	410.60	1,349.24

Cost of Cancer Recurrence						

	Local Disease		12,500.00	12,500.00	12,353.00	12,059.75

	Metastatic Disease		124,767.00	117,714.00	95,525.56	108,325.20

Cost of Cardiac Toxicity			2,015.20	3,214.00	759.60	322.00

**Total Incremental Cost (S$)**			**70,376.73**	**62,769.82**	**35,462.94**	**51,040.63**

**Incremental Costs in Singapore**		**S$ 50,512.63**	**US$ 24,130.16**	**(2009)**		

		**S$ 44,879.82**	**US$ 26,971.05**	**(2005)**		

Notes to Table 1: PMC, private medical centre. GRH, government restructured hospital. HER2neu testing included costs of immunohistochemistry for all patients and fluorescence in-situ hybridization for 30% of patients. There were 12 extra visits with oncologist. Cardiac monitoring included 4 tri-monthly echocardiograms. Cost of local recurrence included mastectomy and hospital stay. Standard of care for patients who progressed on trastuzumab included (1) capecitabine + lapatinib, (2) capecitabine + trastuzumab and (3) trastuzumab + lapatinib. The estimate assumed that patients received 9 months of treatment with (1) or (2) followed by 3 months of treatment with (3). Excess Local recurrence without trastuzumab is estimated at 1% and estimated distant recurrence at 16% based on clinical trials. Cost of treatment of Cardiac toxicity was 4% × cost of 3-monthly cardiology visits + medications for 1 year

The weighted average of incremental direct costs were S$ 50,512.63 in 2009 Singapore dollars and US$ 34,130.16 in 2009 United States dollars. To allow for international comparison and to comply with current comparative effectiveness guidelines these values were S$ 44,879.86 and US$ 26,971.05 in 2005 dollars.

Note that the largest ticket in all hospitals was the cost of the drug which accounted for more than overall costs before they were discounted by the benefits of avoided recurrences. One should also mention that the lowest cost provider charged patients nearly half of what the highest cost provider did.

### Indirect Costs

Indirect costs were calculated as productivity lost during treatment and the cost of transportation to receive treatment. Indirect incremental costs associated with the use of trastuzumab in Singapore were 2005 US$ 932.97.

### Indirect Benefits

Indirect benefits were determined as productivity gained based on the QALYs saved with trastuzumab in the treatment of early breast cancer in Singapore. They are depicted for each of the previously discussed datasets.

#### Indirect Benefits Based on Liberato et al Dataset

In this model, chemotherapy alone yielded an increase of 8.03 QALYs compared to 9.22 QALYs when trastuzumab was added. As such incremental indirect benefits in productivity (discounted annually at 3%) were the difference between the extra productivity achieved in 9.22 years (2005 US$ 243,847.69) minus that achieved in 8.03 years (2005 US$ 216,023.08), namely 2005 US$ 27,824.60.

#### Indirect Benefits Based on Kurian et al (2007) Dataset

In Kurian's model treatment with trastuzumab generated 10.77 QALYs versus 9.35 QALYs with chemotherapy alone. As such, incremental indirect benefit was calculated as 2005 US$ 31,898.05.

Indirect Benefits Based on Garrison et al (2007) Dataset.

Finally, Garrison et al calculated benefits of 11.78 and 10.08 QALYs with and without trastuzumab, yielding incremental indirect benefit of 2005 US$ 37,167.08.

### Societal Costs (Benefits) Associated with the Use of Trastuzumab in Singapore

With the data presented above one can calculate the societal costs (benefits) of trastuzumab in the treatment of early HER2neu-positive breast cancer in Singapore for each of the dataset as follows: **Societal Costs (Benefits) = Direct Costs + Indirect Costs - Indirect Benefits**. (Remember that indirect benefits are already accounted in direct costs as seen in Table 1).

To account for variation and assess the precision of this analysis, 95% confidence intervals were also calculated based on the 95% confidence interval of the QALYs derived from each model. These data are represented in Table 2.

**Table 2:** Societal Costs (Benefits) Associated with Trastuzumab in the Treatment of HER2neu-positive Early Breast Cancer in Singapore, the United States and Italy

Model		Cost/QALY 2005 US$	Cost (Benefit) 2005 US$	95% CI 2005 US$
**Singapore Setting (This study)**				

	Liberato et al	22,664.75	79.42	16,854.97 to (16,353.79)

	Kurian et al	18,993.70	(3,944.03)	11,295.76 to (22,213.99)

	Garrison et al	15,865.32	(9,263.06)	(227.30) to (12,274.98)

				

	Sensitivity Analysis			

		Lowest Range	10,685.78	

		Highest Range	(17,298.79)	

**United States Setting**				

	Liberato et al	18,970.00	10,834.07	12,797.05 to (33,982.92)

	Kurian et al	39,982.00	11,729.38	14,901.69 to 7,038.57

	Garrison et al	26,417.00	n/a	n/a

**Italian Setting**				

	Liberato et al	11,984.67	(1,287.63)	14,958.42 to (17,116.39)

Notes to table 2. Values are presented in 2005 US$ (unless otherwise stated) and represents costs (no parenthesis) or benefits (within parenthesis). Societal costs and benefits derived from reference 6. n/a, not available. See text for further details.

#### Based on Liberato et al

Assessment of societal costs (benefits) showed a cost of 2005 US$ 79.42 per patient associated with the use of trastuzumab in Singapore. The 95% confidence interval varied from a cost of US$ 16,854.97 to a benefit of US$ 16,353.79 per patient treated. Cost per QALY was 2005 US$ 22,664.75.

#### Based on Kurian et al

This model yielded a societal benefit of 2005 US$ 3,944.03 per patient treated with a 95% confidence interval ranging from a cost of US$ 11,295.76 to a benefit of US$ 22,213.99. Cost per QALY was 2005 US$ 18,993.70.

#### Based on Garrison et al

Finally, on the third model, society benefits were 2005 US$ 9,263.06 per individual treated, ranging from US$ 227.30 to US$ 12,274.98. Cost per QALY was 2005 US$ 15,865.32.

### Sensitivity Analysis

At the lowest range of the sensitivity analysis, using the highest cost, from private medical centre number 1 and the lowest QALYs gained (as in the model by Liberato et al), treatment with trastuzumab in Singapore costs 2005 US$ 10,685.78 per patient. At the highest range, using cost data from government restructured hospital 1 and the benefits in the model by Garrison et al, treatment of early HER2neu-positive breast cancer in Singapore yields a benefit of 2005 US$ 17,298.79 per individual treated.

## Discussion

In summary, based on the current study, treatment of early HER2neu-positive breast cancer with trastuzumab is associated with a small cost or a significant benefit depending on the model used. Using the Liberato et al dataset this paper described a cost of US$ 79.42 per patient treated, contrasting with the larger benefits seen with the models by Kurian et al (US$ 3,944.03) and Garrison et al (US$ 9,263.06).

Moreover, the variance seen in the 95% confidence intervals and sensitivity analysis is relatively narrow, ranging from a highest societal cost of 2005 US$ 16,854.97 and a highest societal benefit of 2005 US$ 22,213.99 per individual treated. While these results suggest that the use of trastuzumab leads to societal benefits and support payers in providing this treatment, this paper cannot rule out that the targeted agent might actually lead to higher costs than benefits in Singapore. The following discussion will review the weakness and strengths of this study and will provide a conclusion and recommendations aiming to increase the potential societal benefits of trastuzumab in the island-nation.

Health care in Singapore is financed by a mix of compulsory health-savings accounts (Medisave), an opt-out catastrophic insurance (Medishield) and government provision for patients without means (Medifund). Moreover, the government provides means-based subsidies that can reach up to 80% of hospital expenditures. Public and private service providers compete to provide affordable and world-class medical services [16].

If not well planned, however, the limits on deduction built in the Medisave system can lead to rationing and not rationalization of medical care. As such, health economics analysis of new technologies such as trastuzumab should be undertaken to determine the most efficient level of deduction for individual's Medisave accounts and to assist patients in understanding if there is an individual and societal economic benefit in receiving the drug.

The first and major weakness of this study resides in the use of previously published cost-effectiveness studies instead of the development of an independent cost-effectiveness model in Singapore. While one might argue that this diminishes the applicability of this analysis, as the current available clinical data is based on North-American and Europe, a locally developed model would still have health outcomes similar to the previously published ones. Indeed the QALYs saved in the model by Liberato [7] were identical in the United States and Italian settings. Moreover, the use of 3 datasets - those by Liberato, Kurian and Garrison and their colleagues [7-9], increases the range of possible results, strengthening the applicability of this analysis as the data presented are homogenous and consistent.

The use of GNP per capita as a measure of productivity can also be criticized as it is not age and gender specific, introducing a possible source of bias. Moreover, actual median wages in Singapore are lower than GNP per capita further confounding the issue. Finally, it does not take into account if a patient is employed - and therefore productive. Despite these shortcomings, the availability of GNI per capita data for several nations allows for cross-country comparison and may aid in decision-making as it is a measure commonly known by policy makers.

A third weakness - or actually series of weaknesses - has more to do with health economics assessments in general than with this paper in particular. Methods based on incremental costs often fail to account for the resources used on standard therapy (in this case the costs of local treatment, mastectomy and radiation therapy, and systemic treatment with chemotherapy), and might underestimate the actual resources employed.

Within the same group of limitations, one must note that health economic models extrapolate long term horizons from the short term data available from clinical trials. As such, the benefits may become more or less favourable over time, introducing another source of bias. Further more, clinical trials have strict inclusion criteria, making it harder to extrapolate data to usual populations seen in clinical practice.

Moreover, there are ethical issues that are beyond the scope of this paper but involve the difficulty of assigning a monetary value to a human life.

National settings are also important in the definition of cost-effectiveness. In the United Kingdom, the National Institute for Clinical Excellence [17] deems a new technology cost-effective if the cost per QALY saved is 30,000 British pounds or less; while in the United States (where no agency is actually responsible for setting cost-effectiveness standards) physicians and policy-makers usually accept treatments that cost up to US$ 150,000 or more per life year saved [18]. The World Health Organization considers interventions that are less than the national income per capita as very cost-effective [19]).

In this paper, one assumes that interventions which generate economic benefits should be undertaken while those that generate costs should be rejected, bringing us back to the ethical issue of placing a monetary value on a human life. This is one of the strengths of this study when compared to the cost-effectiveness analyses conducted by Liberato, Kurian, Garrison and their colleagues. The societal perspective in this analysis allows one to conclude that trastuzumab is likely to generate economic benefits in the treatment or early HER2neu-positive breast cancer in Singapore.

One of the study's strength is its use of charges that each medical centre would impose to a patient with the base case characteristics, through a survey of 4 centres which together attend to 60-70% of patients with cancer in the island-nation. Most economic analyses use published cost data such as those from the Centres for Medicare and Medicaid in the United States [20], potentially misrepresenting actual costs, as they do not usually include overhead and other medical centre indirect costs such as rental, personnel, etc.

Moreover, as mentioned above, using 3 published cost-effectiveness studies to derive the health economic benefits of trastuzumab, gives more strength to the conclusions as one sees the homogenous, consistent results obtained with all 3 models.

Finally, it is interesting to compare the author's prior assessment of the societal costs and benefits associated with trastuzumab in the US and European setting with the current analysis. Based on the data from Liberato et al, trastuzumab led to an incremental benefit of $10,834.07, for which the 95% confidence interval ranged from a cost of $12,797.05 to a benefit of $33,982.92 in the United States setting. The corresponding values for Italy showed a more modest benefit of 2005 US$ 1,287.63, with a 95% CI ranging from a cost of $14,958.42 to a benefit of $17,116.39.

The results based on the study by Kurian et al were slightly different but not unexpected as their estimated costs were higher. The incremental cost with trastuzumab was 2005 US$ 11,729.38 (95% Confidence Interval, $7,038.57 to $14,901.69).

These data contrast to the current study as the costs in Singapore were lower than those in the United States and Italy. Moreover, as the calculated incremental QALYs saved were higher in the models by Kurian and Liberato, these models yielded greater benefits in the current analysis. In the end, however all these results fall within a relatively narrow band that goes from a highest cost of 2005 US$ 16,854.97 to a highest benefit of 2005 US$ 22,213.99

As such, the current study's limitations are offset by its strengths and implications for the provision of health care in Singapore and these data support Liberato, Kurian and Garrison's conclusions that therapy with trastuzumab in the adjuvant setting is cost-effective in developed countries, although it might lead to greater societal costs than benefits. Longer follow-up in the adjuvant trastuzumab studies will help shed light on many of the uncertainties seen in the data presented.

Our greatest challenge, however, is to broaden the reach of these technological improvements both in underserved populations in developed countries and in less affluent nations. It is important that the medical oncology community continues to promote societal discussion on health economics topics, especially on the issue of treatment access and affordability.

For now, civil society has helped increase access to the drug in the island-nation and the government is broadening its actions. The Singapore Cancer Society, a charity funded by private donations, helps provide subsidies ranging from 25 to 75% of total drug costs to many needy patients and, recently, the Singapore government disclosed that it will include trastuzumab in a new medication assistance fund.

But we need to and can do better.

Available policy options range from government intervention - which has its own set of problems - to more market-oriented approaches (which the author favours). For a lengthier discussion see reference [21]. In the specific case of Singapore, two sensible changes might suffice to increase utilization of this clinically active and cost-effective agent.

First, price discrimination (also known as tier-pricing) should be employed and Roche (which now holds the rights to sell trastuzumab) should consider decreasing the price of the drug by approximately 15%-20%. As the cost of trastuzumab is the largest element in the incremental direct costs associated with its use this measure would bring the lowest range of the sensitivity analysis closer to zero increasing the likelihood that society would reap economic benefits with the use of the targeted agent. As it would also bring costs closer to current Medisave and Medishield limits of deduction (approximately S$2,600 per 3-week cycle) it would likely increase revenues for the pharmaceutical company as more patients would be able to afford the drug.

Second, the government, through the ministry of health, could consider tweaking the current Medisave system in order to allow for a greater deduction from each individual's health savings account for the use of trastuzumab in the treatment of patients with early HER2neu-positive breast cancer in Singapore. This measure could also increase the number of patients who would be able to afford it, generating greater benefits to society.

## Conclusion

Treatment with adjuvant trastuzumab is likely to generate net societal economic benefits in Singapore. Nevertheless, the lower range of possible outcomes does not refute the possibility that treatment may actually generate costs. These costs however clearly fall within the usual range of acceptable cost-effectiveness.

In the future, emerging clinical data might provide information that shorter treatment duration is as efficacious as the current year-long course, while achieving greater cost-effectiveness. In fact, a study named FIN-HER demonstrated that the use of trastuzumab for 9 weeks decreases disease recurrence by 50%, similar to the current standard of care [22]. Uncertainty remains however as this was a relatively small study and it did not have a comparator arm with trastuzumab given for a year. Several studies are currently investigating shorter durations of treatment with trastuzumab.

For now, the 2 suggestions described in this paper may provide the best chance that more patients will be able to benefit from trastuzumab in the treatment of early HER2neu-positive breast cancer in Singapore.

## Abbreviations

Abbreviations are described in the text at their first occurrence

## Competing interests

The author is President of the Singapore Chapter of the International Society for Pharmacoeconomics and Outcomes Research and a member of the Executive Committee of the Singapore Society of Oncology in addition to his affiliation with the Johns Hopkins Singapore International Medical Centre and University.

He is solely responsible for the statements in this paper which represents his views and not those of the institutions he is associated with.

Dr. Lopes has received research support from Genentech and Roche, but he has not received any paid honorarium for talks or otherwise from these companies, which make and commercialize Trastuzumab.

## Authors' contributions

GLL designed and conducted the study. He also wrote this report.

## Pre-publication history

The pre-publication history for this paper can be accessed here:

http://www.biomedcentral.com/1471-2407/11/178/prepub

## Supplementary Material

Additional File 1 **Assumptions of each of the models by Liberato et al, Kurian et al and Garrison et al**. Click here for file
