# Supplementary material for: Societal Costs and Benefits of Treatment with Trastuzumab in Patients with Early HER2neu-Overexpressing Breast Cancer in Singapore
Source: BMC Cancer. 2011 May 18;11:178. doi: 10.1186/1471-2407-11-178 (PMC3118893; doi:10.1186/1471-2407-11-178)
Supplement: Additional File 1 — Assumptions of each of the models by Liberato et al, Kurian et al and Garrison et al. [file 1471-2407-11-178-S1.DOC]

Appendix 1. Assumptions for each of the models used:

Assumptions from Kurian et al’s Dataset

This dataset used a Markov model to estimate the long term health outcomes, quality of life and costs from treatment with trastuzumab. Using TreeAge Pro 2005 software the authors generated a Markov state-transition model with a cycle length of a month, extending the time-horizon from the median 2 years presented at the available randomized studies (Miller, 1994).

Results were presented as incremental cost-effectiveness ratios (ICER), in cost per QALY saved; both costs and QALYs were discounted at 3% in the base case. They performed this analysis from a partial societal perspective, as per the recommendations of the Panel on Cost-Effectiveness in Health and Medicine (Gold, 1996).

Their model applied to 49-year-old women with early-stage breast cancer with positive amplification or over-expression of the *HER2*/*neu* oncogene (this was the median age of participants in the published randomized trials). Their analysis applied to women who had undergone surgical resection of all apparent disease, with and without involved axillary lymph nodes, just as in the trial populations (Romond, 2005, and Piccart-Gebhart, 2005).

The authors calculated breast cancer recurrence rates in the first 4 years after the initiation of adjuvant treatment from the analysis of the NSABP B-31 and NCCTG 9831 randomized clinical trials, using the no-trastuzumab control arm. For a longer time-horizon they extrapolated recurrence rates in the no-trastuzumab arm from published data on survival after HER2/*neu* amplified breast cancer (Riou, 2001, Ferrero-Pous, 2000, and Tsuitsui, 2002). For the trastuzumab-containing arms, they calculated recurrence rates in the first 2 years from the relevant clinical trials for each regimen. Beyond that they assumed that the relative risk reduction for breast cancer recurrence would decline, as reported by the Early Breast Cancer Trialists’ Collaborative Group (2002) for chemotherapy. Because there is little evidence that trastuzumab effects vary with age they used the hazard ratios for women younger than age 50 years in their base case, assuming a one-third decrease in the relative risk reduction with both AT arms in years 2 to 4, and an additional one-third decrease in years 5 to 10. They held the values constant beyond year 10.

In sensitivity analyses, they considered a greater decline in benefit, as it is known for chemotherapy in women age 50 to 69 years. The authors used age-specific mortality data from the National Center for Vital Statistics to estimate non–breast cancer death rates (National center for vital statistics, 2009). They converted all rates to monthly health-state transition probabilities.

Reflecting the then current US, and now current worldwide clinical practice, they assumed that all patients received trastuzumab for recurrence of systemic HER2/*neu*-positive breast cancer. Using data from the pivotal randomized trial of trastuzumab for metastatic disease, a median survival of 25 months with 9 months of trastuzumab therapy was assumed.

In the non-trastuzumab arm (NT), patients received doxorubicin 60 mg/m2 plus cyclophosphamide 600 mg/m2 intravenously for four cycles every 3 weeks, followed by paclitaxel administered at 80 mg/m2 intravenously for 12 cycles every week, as in the control arms of theNSABPB-31 andNCCTG9831 clinical trials. In the anthracycline-containing AT arm, patients received the same chemotherapy regimen as in the NT arm, with the addition of trastuzumab to weekly paclitaxel. The initial trastuzumab dose was 4 mg/kg, followed by weekly doses of 2 mg/kg for a total of 1 year of treatment. In the non-anthracycline containing (NAT) arm, patients received docetaxel 75 mg/m2 plus carboplatin at an area under the curve of 6 every 3 weeks for 6 cycles. Trastuzumab was administered weekly concurrently with chemotherapy, at the same doses described above for 1 year.

Per the joint analysis of the NSABP and NCCTG anthracycline-based regimens, the authors assumed that cardiac toxicity, symptomatic and asymptomatic, increased by an absolute rate of 3% in the AAT arm. They assumed that approximately 80% of patients who develop cardiac toxicity after the AAT arm improve, becoming asymptomatic with or without therapy, within 6 months (Tan-Chiu, 2005). Consistent with reports of the clinical trials, they assumed that the rate of cardiac deaths was not increased in the AAT arm. In a sensitivity analysis, they assumed that the risk of cardiac death increased to levels reported with chronic cardiac systolic dysfunction. They assumed that cardiac toxicity was not elevated in the NAT arm, per results of the preliminary BCIRG 006 analysis (Slamon, 2005). They did not explicitly model non-cardiac adverse events as these did not vary significantly between regimens in the randomized clinical trials.

Previously published adjustments for quality of life associated with adjuvant chemotherapy, cardiac toxicity, and breast cancer recurrence, assigning a utility of 0.85 to adjuvant chemotherapy were used (Hillner, 1991 and 1992). Other assigned utilities were 0.64 to symptomatic heart failure and a utility of 0.55 to metastatic breast cancer (Hutton, 1996). They assigned no utility decrement to AT, but included the cost of time lost from work because of AT treatment in the total cost of trastuzumab (United States Bureau of Labor Statistics, 2009). They also adjusted the utility associated with life in the disease-free state for age, estimating from time trade-off assessments reported in the Beaver Dam Health Outcomes Study (Fryback, 1993).

Costs of AT included costs of the drug, drug infusion, monthly oncologist visits, and cardiac monitoring with echocardiogram or multigated acquisition scan every 3 months during therapy (as per clinical trial protocols - Romond, 2005). Total costs of adjuvant chemotherapy without trastuzumab, including supportive medications and other supportive care, were estimated from a publication which used Regence Blue Shield claims data linked with the Cancer Surveillance System of the US Surveillance, Epidemiology, and End Results database to evaluate costs of adjuvant chemotherapy for breast cancer according to age, stage, type of surgery, and comorbid conditions. For the docetaxel and carboplatin regimen, they added the difference between the cost of these agents and those in the NT arm to the total adjuvant chemotherapy cost. They estimated costs of breast cancer recurrence from publications which incorporated costs of chemotherapy, supportive, and end-of-life care. They also added the cost of 9 months of trastuzumab therapy (Kurian, 2007).

Costs of asymptomatic heart toxicity included monitoring with echocardiogram or multigated acquisition scan every 3 months, cardiologist visits, and therapy with a beta-blocker and angiotensin-converting enzyme inhibitor. Costs of symptomatic heart failure were those of asymptomatic left ventricular ejection fraction decrease, plus the cost of an initial 3-day hospital stay (Kurian, 2007). All costs were updated to 2005 US dollars.

As the longer-term effects of therapy are still unknown, they evaluated the impact of changes in recurrence rates under each treatment strategy using probabilistic sensitivity analysis. Base case recurrence rates were varied within the 95% CIs reported in the AT randomized clinical trials. Rates under each strategy were varied under an independent distribution, through a simulation of a thousand samples. They varied all other variables for which uncertainty existed in one-way sensitivity analyses. Ranges of variation were based on published data whenever available, and otherwise varied within 20%.

In the base case analysis, treatment with the NT regimen yielded 9.35 QALYs at a cost of $133,429, the AAT regimen yielded 10.77 QALYs at a cost of $190,092, and the NAT regimen yielded 10.61 QALYs at a cost of $206,561. Compared with the NT regimen, the AAT regimen yielded an ICER of $39,892/QALY. The AAT regimen dominated the NAT regimen (meaning that it cost less and was more effective).

Assumptions from Liberato et al’s Dataset

Liberato et al designed a decision tree using TreeAgePro software. This tree compared long-term costs and effectiveness of adjuvant chemotherapy plus trastuzumab and chemotherapy alone for patients with HER2-positive early breast cancer. They used a hypothetical cohort which was similar to that enrolled onto the pivotal clinical trials that led to the approval of trastuzumab. Their Markov model simulated the long-term clinical evolution of such patients from the relatively short follow-up observed in the clinical trials, tracking patients’ transitions between mutually exclusive health states that last a fixed length of time. Patients accumulate life-years, quality-adjusted life-years (QALYs), and costs in each cycle. Input probabilities determine the transitions. Five health states of 3-month duration were included. These were disease free, local relapse, disease free after local relapse, metastatic disease, and death.

Patients start in the disease-free state, after completion of the first part of adjuvant chemotherapy. During the first 4 months, patients were allowed to receive paclitaxel alone or the same chemotherapy regimen plus trastuzumab (2 mg/kg/wk), whereas during the subsequent 9 months, only patients assigned to trastuzumab continued to receive treatment. Disease-free patients receiving adjuvant trastuzumab could incur symptomatic or asymptomatic cardiac dysfunction, which assumed interruption of the trastuzumab and temporary changes of both costs and quality of life. Disease-free patients could relapse. Most patients incurring a local relapse were allowed to receive effective treatment and return to a disease-free health status (referred to as disease-free after local relapse) within 12 months, whereas 10% of the patients shifted to a systemic disease status. All of the patients incurring a systemic relapse entered the metastatic disease state and could only move to the death state.

The authors derived transition probabilities and proportions from meta-analyses or randomized clinical trials. The rate of relapse early after adjuvant therapy was derived from the combined report of the NSABP B-31 and NCCTG N9831 trials, which reported the longest follow-up among the randomized trials assessing adjuvant trastuzumab (Liberato, 2005). They assumed that no additional benefit would be accrued after 5 years. Cardiac mortality was assumed to be null.

Long-term outcomes of disease-free patients that could not be derived from available randomized trials were obtained from a large meta-analysis by the Early Breast Cancer Trialists’ Collaborative Group (2002). The long-term outcomes of patients who developed local recurrence were derived from a large cohort study (Liberato, 2005). For distant relapse, chemotherapy and trastuzumab was the treatment if the patient had not received adjuvant trastuzumab. Chemotherapy alone was the treatment for patients who had received prior trastuzumab. The authors also set that patients in any health state might die of non-breast cancer causes, using national mortality rates for females (adjusted according to the patients’ age).

Italian and United States health care systems perspective was used, without estimating indirect costs. The authors also assumed patients weighed 60 kg and had a body-surface area of 1.6m2 to calculate the doses of medications. When considering the costs for cardiologic monitoring, patients were assumed to have a quarterly echocardiography. The cost of management of heart failure was obtained from available literature (Liberato, 2007), as were other costs, such as those of local relapse and metastatic disease. Utilities obtained from the medical literature were used to adjust for quality of life.

Liberato et al entered each rate, cost, and utility into the model along with distributions for age, input costs, probabilities and proportions, and the relative risk of relapse. Both future costs and life-years were discounted at a 3% yearly rate. They conducted a Monte-Carlo analysis with 10,000 simulations and plotted acceptability curves with a 15-year-long time horizon and calculated the incremental cost per QALY. Finally, Liberato et al ran probabilistic sensitivity analysis for all the parameters.

The model calculated that adjuvant trastuzumab resulted in a benefit of 1.34 life-years and 1.54 QALYs. The overall savings achieved by the adjuvant treatment with trastuzumab were about €16,500 ($19,500), but the cost of the adjuvant trastuzumab treatment offset the savings: costs for each patient starting trastuzumab were about €32,000 ($40,000) for the drug, its administration, and the possible occurrence of cardiac dysfunction. Therefore, the incremental cost of adjuvant trastuzumab was €15,476 ($20,211) and the incremental discounted cost effectiveness was €14,861/QALY ($18,970/QALY). The acceptability curve of adjuvant trastuzumab showed that it cost less than €20,000/QALY ($27,000/QALY) with a probability of 91%.

The main results of the probabilistic sensitivity analysis are not shown but can be seen in Liberato’s original paper (2007).

Assumptions from Garrison’s et al Dataset

Garrison et al conducted a cost-utility study which assessed costs and outcomes of 2 different treatment options, using a payer and a partial societal perspective. As in the other 2 models that make the basis of the benefits in this paper, the based their analysis on the results of the NSABP B-31 and NCCTG N9831 clinical trials. A Markov model with 4 health states – treatment, disease-free, distant recurrence and death – was used to evaluate the expected costs and quality life years saved over a long time horizon (extrapolated from the short term follow-up available from both trials). Garrison et al did not explicitly defined local recurrence as part of their costs. Heart-related adverse events were assumed to be temporary and not to have any direct mortality impact

The base-case estimate was a ‘‘typical’’ patient (their words), namely a 50-year-old woman. Direct costs were based on 2006 Medicare reimbursement rates and other readily available data (Garrison, 2007) and were expressed in 2005 US dollars. Differently than Liberato et al and Kurian et al, the authors also included costs for Her2Neu testing. Both immunohistochemistry (IHC) and FISH tests were included (with 30% of tests being FISH) Average wholesale price was used to estimate drug cost per patient. The authors assumed that 19.9% of patients stopped treatment with trastuzumab earlier than planned. Cardiac function monitoring was monitored with 4.7 multigated acquisition scans or echocardiogram. Significant cardiac events were assumed to be 2.9%.

The authors included incremental indirect costs related to trastuzumab, estimated as time and expenses for travel and medical visits. The average hourly compensation rate from the US Bureau of Labor Statistics (2009) was used to calculate time lost and travel costs were assessed using internal revenue services business travel rates (United States Department of Treasury, 2009).

The authors made adjustments for quality of life in the 4 different health states. Quality-adjusted life years (QALY) were used to express the benefits derived from treatment with trastuzumab. Costs and outcomes were discounted at 3%. Assumptions were made about the Markov transition probabilities for the period beyond actual follow-up in the clinical trials. Recurrence rates were estimated using the Early Breast Cancer Trialists’ Collaborative Group report (2002). Background mortality was based on the standard mortality rate. They assumed that patients in both treatment arms would receive trastuzumab after recurrence. The authors also conducted sensitivity analyses to increase the robustness of the results (data not shown on this manuscript).

The model by Garrison et al determined that treatment with trastuzumab cost an additional US$44,923 and had an expected gain of 1.70 QALYs.
